# Effect of Parkinson’s disease and two therapeutic interventions on muscle activity during walking: a systematic review

**DOI:** 10.1038/s41531-020-00119-w

**Published:** 2020-09-09

**Authors:** Aisha Islam, Lisa Alcock, Kianoush Nazarpour, Lynn Rochester, Annette Pantall

**Affiliations:** 1 Translational and Clinical Research Institute, Newcastle University, Newcastle upon Tyne, UK; 2 School of Engineering, Newcastle University, Newcastle upon Tyne, UK; 3 Biosciences Research Institute, Newcastle University, Newcastle upon Tyne, UK; 4 The Newcastle upon Tyne Foundation Trust, Newcastle upon Tyne, UK

**Keywords:** Parkinson's disease, Biophysical models, Parkinson's disease, Diagnostic markers, Neurophysiology

## Abstract

Gait deficits are a common feature of Parkinson’s disease (PD) and predictors of future motor and cognitive impairment. Understanding how muscle activity contributes to gait impairment and effects of therapeutic interventions on motor behaviour is crucial for identifying potential biomarkers and developing rehabilitation strategies. This article reviews sixteen studies that investigate the electromyographic (EMG) activity of lower limb muscles in people with PD during walking and reports on their quality. The weight of evidence establishing differences in motor activity between people with PD and healthy older adults (HOAs) is considered. Additionally, the effect of dopaminergic medication and deep brain stimulation (DBS) on modifying motor activity is assessed. Results indicated greater proximal and decreased distal activity of lower limb muscles during walking in individuals with PD compared to HOA. Dopaminergic medication was associated with increased distal lower limb muscle activity whereas subthalamic nucleus DBS increased activity of both proximal and distal lower limb muscles. Tibialis anterior was impacted most by the interventions. Quality of the studies was not strong, with a median score of 61%. Most studies investigated only distal muscles, involved small sample sizes, extracted limited EMG features and lacked rigorous signal processing. Few studies related changes in motor activity with functional gait measures. Understanding mechanisms underpinning gait impairment in PD is essential for development of personalised rehabilitative interventions. Recommendations for future studies include greater participant numbers, recording more functionally diverse muscles, applying multi-muscle analyses, and relating EMG to functional gait measures.

## Introduction

Parkinson’s disease (PD) is a multisystem neurodegenerative disease with characteristic features present in both non-motor and motor domains^1^. The non-motor clinical manifestations include sensory impairments such as pain and tingling, depression, hyposmia and altered executive function^2^. The main motor symptoms are resting tremor, bradykinesia, rigidity, postural instability and gait disturbance^3^. This review is concerned with gait dysfunction and therefore will focus on gait and related motor symptoms.

Gait disturbance is characterised by slow shuffling steps^4^, asymmetry^5^ and high stride-to-stride variability^6,7^. The increased energy expenditure associated with dysfunctional gait makes even a short walk a major physical effort^8^, thereby restricting mobility which impacts on quality of life. Fall risk is higher in people with PD^9,10^, which imposes a social and economic burden through hospitalisation^11^ and subsequent health care costs^12,13^. Dopaminergic treatment may reduce some abnormal gait features such as bradykinesia and rigidity^14^. However, other characteristics such as gait instability may not respond to dopaminergic therapy in some people with PD due to various factors as outlined in the review by Nonnekes et al.^15^. Long term treatment is confounded by levodopa-induced dyskinesia, alongside fluctuations in motor response which result in the ‘ON’ and ‘OFF’ states^16^. Consequently, there is an urgent need to develop novel rehabilitative approaches to gait dysfunction in PD.

Optimal gait is dependent upon the functional integration of motor activity at multiple levels. At the micro level, motor units are recruited according to the ‘size principle’ to ensure graduated contraction and consequentially smooth movement^17,18^. At the macro level, timings of muscle contractions between synergists, antagonists and muscles acting on ipsilateral and contralateral joints are precisely regulated. This results in an energy efficient, forward propulsion of the individual’s centre of mass whilst maintaining dynamic stability. Complex neuronal networks orchestrate these constantly fluctuating muscle activation patterns. Sensorimotor integration is a key component underpinning effective locomotor neuronal networks. However, in PD, sensorimotor processing is impaired with resultant changes in motor activity patterns during gait^19^.

**Fig. 1:**
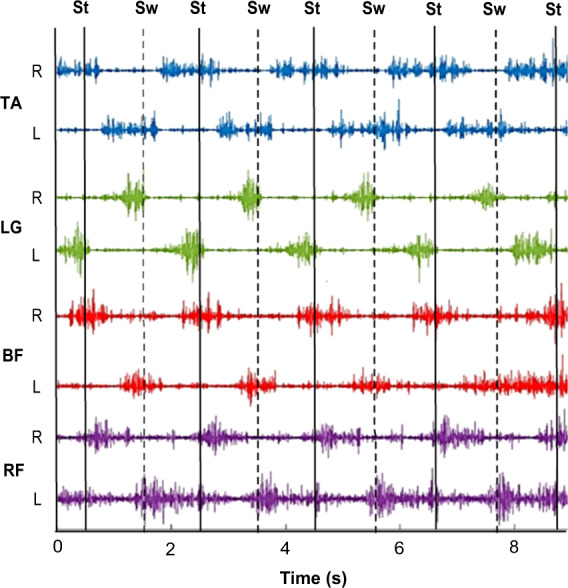
Typical surface EMG signals of four bilateral lower limb muscles recorded from a healthy older adult during walking. Bandpass filtered unrectified EMG signals for tibialis anterior (TA), lateral gastrocnemius (LG), biceps femoris (BF) and rectus femoris (RF) for the right (R) and left (L) legs. Onset of the stance (St) phase of walking for the right leg is indicated by solid vertical lines. Onset of the swing (Sw) phase of walking for the right leg is indicated by dashed vertical lines.

Muscle activity is generally quantitatively assessed by surface or intramuscular electromyography (EMG) which records voltage changes in muscle fibres following stimulation by α-motoneurons. Typical surface EMG signals in a healthy older adult (HOA) of four bilateral lower limb muscles during walking are shown in Fig. 1. Tibialis anterior (TA), biceps femoris (BF) and rectus femoris (RF) are active during the initial loading phase of stance with their corresponding contralateral muscles 180° out of phase. Lateral gastrocnemius (LG), an ankle plantar flexor, is important later in the stance phase for push-off of the foot. TA, an ankle dorsiflexor, is essential for foot clearance during the swing phase. BF contracts again towards the end of the swing phase, decelerating the forward moving leg prior to foot touchdown. Underpinning the timings and magnitude of EMG signals are neural networks. Different characteristics of the EMG signal therefore offer insight into the neural control of locomotion both at the micro and macro level of motor control^20,21^. Relative timing of motor activity onset may reveal dysfunction of sensorimotor integration. Spectral characteristics of the EMG signal may indicate altered motor unit recruitment strategies^22,23^ and presence of fatigue^24^. Multi-muscle EMG analysis and intermuscular coherence may provide information about global control networks^25,26^.

Studies have found characteristic EMG gait patterns in specific populations. Schmitz et al.^27^ have reported that HOAs, compared to healthy young controls, displayed greater LG and TA activity during stance and greater coactivation of muscles acting on the ankle joint with larger differences observed in uniarticular muscles such as soleus and vastus lateralis^27^. Another study observed people with diabetic neuropathy to have earlier onset of activity of soleus (SO), medial gastrocnemius (MG) and semimembranosus/semitendinosus compared to HOA^28^. Alterations in muscle activity patterns during walking have also been observed in people with transfemoral amputation^29^ and individuals with cervical spondylotic myelopathy compared to healthy controls^30^. However, it is not clear what changes occur in muscle activity during walking in people with PD.

Two cardinal motor features of PD likely to leave an imprint on EMG patterns during gait are rigidity and postural instability. Baradaran et al.^31^ observed that rigidity was associated with changes in cortical/subcortical connectivity including the supplementary motor area to the putamen together with increased excitability of the motor cortex^31^. One possible functional consequence of this is greater cocontraction of agonist/antagonist muscle groups and less effective recruitment of individual muscles. Manifestation of gait dynamic instability, defined as instability transitioning from one gait phase to another, may be represented by double-support time^32^ and greater variability of the timing of gait such as stride time^33,34^. A neural correlate of gait variability is the posterior putamen which is associated with automatic movement and exhibits dysfunction in people with PD^35–37^. Gait variability must necessarily be reflected in variability of EMG signals. EMG parameters may provide a better indicator of neurological dysfunction than current widely used gait parameters.

A limitation of the common gait features extracted from body-worn sensors such as inertial measurement units (IMUs), foot switches, and insole pressure sensors is they lack specificity to PD^38^. EMG signals differ from kinematic and kinetic features as they are directly linked to the nervous system, via the α-motoneurons. Several studies have reported differences in features of EMG during non-gait motor tasks in people with PD compared to HOAs^39–41^, from which mechanisms of motor control dysfunction in PD have been inferred. Gait dysfunction is a common motor symptom in people with PD, therefore patterns of EMG during gait are expected to differ in people with PD compared to controls. Gait EMG may therefore be a useful tool in detection of PD; however, little information is available about using gait EMG as a biomarker in PD^42^.

Interpretation of EMG activity is challenging, as there is high intra-individual and inter-individual variability in EMG activity patterns compared to kinematic and kinetic signals^29^. This is due to numerous muscles performing similar actions across joints, resulting in multiple sets of muscles able to perform a specific motor task rather than a single set, described by Latash^43^ as the ‘principle of abundance’^43^. Further compounding the difficulty is the variability of motor symptoms, both at the intra-individual level and inter-individual level. Factors affecting type and severity of motor symptoms include age, the PD phenotype, the stage of PD, type and dosage of medication, responsiveness to medication, and timing of assessment in relation to medication intake^44,45^.

Interventions targeting gait dysfunction in PD must necessarily modify muscle activity to achieve changes in gait kinematics and kinetics. Levodopa is the first line of treatment recommended for targeting motor symptoms in the early stages of PD^46^. Deep brain stimulation (DBS) is recommended for patients with advanced PD whose symptoms are not alleviated by pharmaceutical therapy (41). Krack et al.^47^ have reported improvements in motor function and activities of daily living in patients with PD treated with DBS over a five-year period^47^. Several studies observed DBS and levodopa-induced comparable improvements in gait parameters such as gait velocity^48–51^, step and stride length^49–52^, peak of moment and power at hip and ankle^49,53,54^, and a reduction in double-support time which suggests an improvement in balance and stability^52^. Levodopa and DBS can be administered individually, but the combination of both treatments have demonstrated a greater improvement in gait parameters, possibly due to working synergistically^50,52^. However, there are differential effects of DBS and levodopa on gait (for a review see ref. ^55^), and it is unclear how modification of gait parameters links to the underlying neuromuscular changes.

Understanding the neural mechanisms related to gait dysfunction is essential to improve the effectiveness of interventions, in addition to determining what aspect of the intervention is particularly beneficial. Crucial information regarding the mode of action of therapeutics on neuromuscular control and corresponding kinematics can be obtained by assessing whether they target individual muscles, groups of muscles at the network level, or affect coordination between muscles and limbs. This review will examine the effect of dopaminergic medication and DBS on muscle activity and function.

An essential element of this review is assessing the quality of the studies in terms of external and internal validity. The EMG signal is an indirect measure of muscle activity containing not only the physiological signal but also considerable noise and artefact. It is therefore vital that the EMG signal has been appropriately recorded and processed according to recommended guidelines^56^.

This review systematically investigates studies that have analysed EMG of lower limb muscles during walking in individuals with PD and HOA. The first aim of this review is to critically evaluate PD-related changes in EMG features during walking. A further aim is to examine the effect of dopaminergic medication and DBS on EMG activity. Understanding how muscle activity contributes to gait impairment in PD and effects of interventions is necessary for the development of personalised, evidence driven rehabilitation techniques and to identify biomarkers which may detect early PD^57^.

## Results

### Search yield

**Fig. 2:**
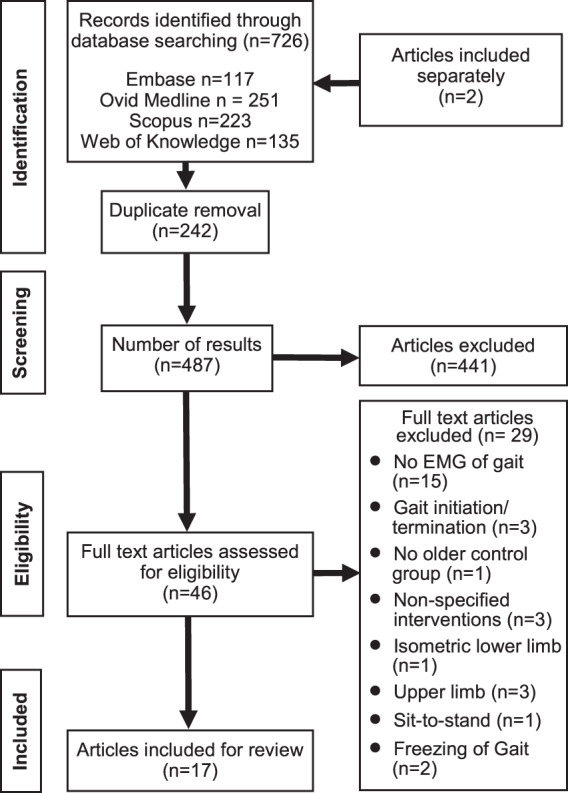
PRISMA diagram presenting overview of the search strategy. The flow chart illustrates how publications were identified and the resulting 17 articles extracted following screening.

The search strategy yielded 726 studies (Fig. 2) of which 242 duplicates were removed. Studies were screened for titles and abstracts and 46 articles were retrieved for full-text screening. Data were extracted from 17 papers with two papers^58,59^ reporting on the same study.

### Quality assessment

**Fig. 3:**
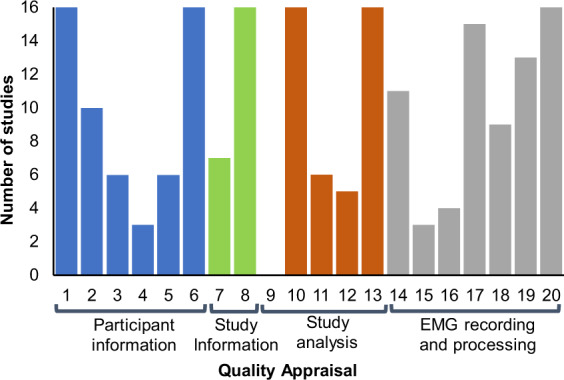
Quality appraisal of the 16 studies reviewed. Number of studies from a total of 16 that scored for each of the 20 questions on the quality appraisal form corresponding to external validity, study information, study analysis and EMG recording and processing techniques.

Table 1 lists the overall scores derived from the quality appraisal form (see Supplementary Information) which ranged from 35% to 90% with a median score of 61%. Figure 3 depicts the number of studies scoring for each of the 20 questions. No studies scored on Q9, which related to justification of sample size and only three studies discussed sampling methods (Q9) or attachment of electrodes. Fewer than half of studies clearly outlined their hypotheses (Q7). All studies described patient characteristics (Q1), aims (Q6), main outcomes (Q8), main findings (Q10) and (Q13) validated outcome measures (Q20).

**Table 1:** Percentage score for each study derived from quality appraisal form (see Supplementary Information).

Studies	Score (%)
Albani et al.^66^	35
Arias et al.^67^	80
Bello et al.^73^	85
Dietz et al.^63^	50
Dietz et al.^58,59^	60
Jenkins et al.^62^	65
Miller et al.^69^	55
Mitoma et al.^65^	40
Rodriguez et al.^70^	60
Rose et al.^72^	90
Caliandro et al.^71^	65
Cioni et al.^64^	55
Ferrarin et al.^68^	58
Pourmoghaddam et al.^60^	61
Rizzone et al.^54^	55
Roemmich et al.^61^	61

### Study protocol

Sample size ranged from nine^60,61^ to forty^62^ for individuals with PD and from seven^63^ to forty^62^ for healthy aged matched controls. Ages ranged from 58.3 ± 13.5 years^64^ to 76 ± 6 years^60^ for individuals with PD and from 58.0 ± 7.6 years^64^ to 74.4 ± 5.8 years^65^ for HOA. A greater proportion of males were assessed for the PD groups. Eight studies did not report on gender^58,59,63,66–70^.

**Fig. 4:**
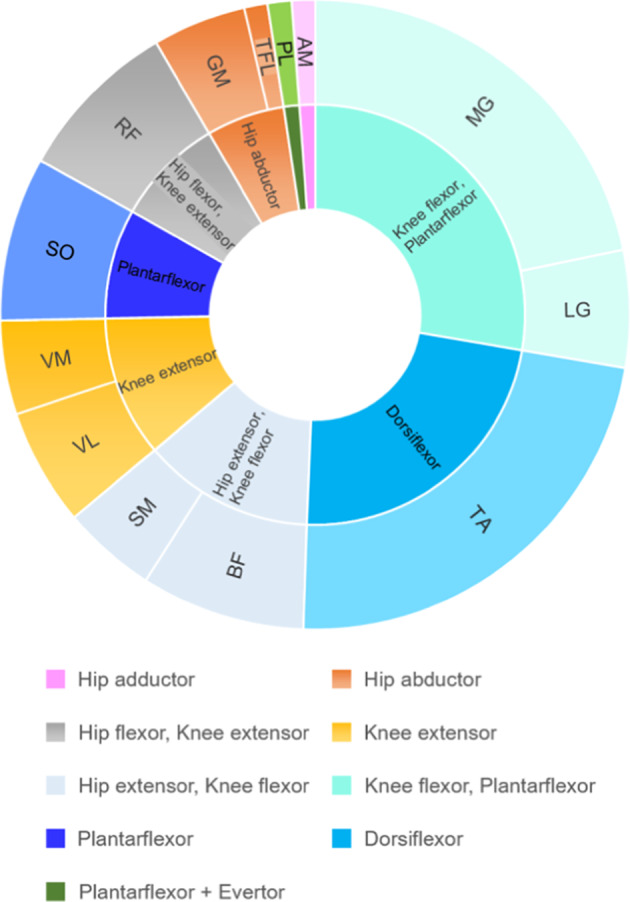
Proportion of studies recording lower limb muscles and muscle groups. The chart is normalised to 100% of studies included in this review. The outer ring contains recorded muscles: adductor magnus (AM), biceps femoris (BF), gluteus medius (GM), lateral gastrocnemius (LG), medial gastrocnemius (MG), semimembranosus (SM), semitendinosus (ST), rectus femoris (RF), tibialis anterior (TA), vastus lateralis (VL), vastus medialis (VM). The inner ring contains the functional muscle group.

Thirteen lower limb muscles were recorded in the reviewed studies with knee flexors and ankle plantarflexors being most frequently recorded^60–65,67,70^ and only one study measuring hip adductors^65^ (Fig. 4). Fourteen studies measured muscles bilaterally^54,58,59,61–72^, one study assessed only the right leg^60^ and one study the most affected leg^73^.

The EMG recording sessions were all restricted to a gait laboratory. Walking surfaces included level over ground walkways^54,62,64–71,73^ of lengths between 6 m^62,65,67^ and 25 m^73^, motorised treadmills^60,66,73^, split belt treadmills^61,63,70^, positive pressure treadmill^72^, a treadmill simulator^73^ and body unloading over a treadmill^58,59^ (Tables 2, 3). A range of walking speeds were investigated including twelve studies at self-selected comfortable walking speed^54,60–62,64,65,67–71,73^ (Tables 2, 3). In the remaining studies, treadmill speeds were set at 0.25–1.0 m/s^63^, 0.34 ± 0.14 m/s^58,59^, 0.83 m/s^72^ and 1.5 m/s^66^.

**Table 2:** Aims, protocol and key findings of non-intervention studies.

Study	Aims	Participant characteristics	Medication	Walking surface	Walking task	Key findings
Albani et al.^66^	Evaluated the relationship between freezing of gait (FoG) in PD and no FoG and EMG patterns during treadmill walking.	PD (*n* = 10) Age: 64 ± 13 Gender: not reported H&Y: 3–4 (FoG) 1.5–2.5 (no FoG) UPDRS-III: 53.2 ± 14.7 (FoG) 24.8 ± 6.5 (no FoG) HOA (*n* = 7) Age: 63 Gender: 4m/3f	ON	Treadmill	Walking for 60 s at belt speed of 0.3 m/s and 1.5 m/s.	Decreased left MG amplitude during stance in the PD groups compared to HOA. Greater left TA activity during swing in the PD groups compared to HOA at slow walking speed.
Arias et al.^67^	Investigated the effect of walking speed on muscle coactivation and differences between healthy adults and PD groups.	PD (*n* = 20) Age: 68.3 ± 6.9 Gender: not reported H&Y: 3–4 HOA (*n* = 20) Age: 66.6 ± 7.8 Gender: not reported HYA (*n* = 7) Age: 21.9 ± 1.5 Gender: not reported	ON or OFF	Overground 6 m walkway	Four trials of walking at self-selected speed followed by fast walking (FW). Walking to a metronome set at 50–110% of FW cadence.	No association between coactivation index and gait speed was reported. High variability of coactivation between individuals. No difference between usual walking and walking to a metronome.
Bello et al.^73^	Compared differences in EMG between PD and HOA. Differences in EMG between overground, treadmill and a treadmill simulator walking in PD.	PD (*n* = 9) Age: 71.0 ± 6.0 Gender: 8m/1f H&Y: = 3 UPDRS-III: 38.7 ± 7.3 HOA (*n* = 9) Age: 71.0 ± 8.6 Gender: 8m/1f	ON	Overground 1.3 m walkway Treadmill walking Treadmill simulator walking	Three trials of walking at self-selected walking speed. Three minutes walking overground on the treadmill at self-selected speed. Three minutes walking on a treadmill simulator.	Decreased activity of TA for load phase (PD and HOA). Lower coactivation of BF/VL for swing phase (PD and HOA) and single support (PD only). Lower coactivation of TA/GM for single support (PD and HOA).
Dietz et al.^63^	Evaluated EMG of the lower limb during various speeds of treadmill. Investigated interlimb coordination by varying split-belt treadmill conditions.	PD (*n* = 14) Age: 61 ± 11.4 Gender: not reported Movement disorder: 1–2 (scale not stated) HOA (*n* = 10) Age: 60.6 ± 6 Gender: not reported	ON	Split-belt treadmill	Walked on treadmill at speeds of 0.25, 0.5, 0.75 and 1.0 m/s. Various combinations for both legs for 60 s per condition.	Greater coactivation in PD compared to HOA, independent of treadmill walking condition. Less modulation of muscle activity, particularly for MG. Longer double support in PD. Decreased ability of PD to change stride frequency with treadmill speed.
Dietz et al.^58,59^	Investigated the effect of body unloading on lower limb EMG during treadmill walking.	PD (*n* = 11) Age: 63.4 ± 12.7 Gender: not reported H&Y: 1.5–3 UPDRS: 27.3 ± 9.4 HOA (*n* = 7) Age: 63.0 ± 6.5 Gender: not reported HYA (*n* = 9) Age: 26.8 ± 3.3 Gender: not reported	ON	Treadmill	Walked on treadmill at a speed of 0.34 ± 0.14 m/s for each body unloading condition.	MG and RF activity decreased during unloading. MG was less sensitive to unloading in PD. TA and BF activity showed minimal change during unloading.
Jenkins et al.^62^	Examined effects of increasing plantar cutaneous sensation with a ribbed insole on EMG and gait parameters.	PD (*n* = 40) Age: 65.4 ± 8.0 Gender: 24m/16f H&Y: 1–3 UPDRS-III: 22.6 ± 8.4 HOA (*n* = 40) Age: 64.7 ± 7.7 Gender: 15m/25f	ON	Overground 6.1 m walkway	Ten walking trials at self-selected walking speed under two conditions: 5 with ribbed insole 5 with conventional flat insole.	In PD, TA peak activity (loading phase) occurred later than in HOA. The effect of a ribbed insole resulted in earlier peak activation of TA (loading phase).
Miller et al.^69^	Investigated effect on EMG symmetry and variability following a 3-week RAS gait training programme. Comparisons made between individuals with PD and HOA.	PD (*n* = 18) Age: 71 ± 8 Gender: not reported H&Y: 2–3 HOA (*n* = 19) Age: 68 ± 7 Gender: not reported	ON	Overground 8 m walkway	Two trials of walking at self-selected walking speed. Recordings taken before and after 3-week RAS intervention.	No RAS: PD showed greater shape variability of TA and MG vs HOA. TA displayed the greatest shape variability in both groups. Phase variability for MG was smaller in PD vs. HOA. Higher asymmetry of TA and MG was reported in PD. RAS: Walking speed increased MG and TA variability and TA asymmetry decreased in PD. In HOA, no significant changes were reported.
Mitoma et al.^65^	Compared EMG and kinematics in individuals with PD to HOA during walking.	PD (*n* = 16) Age: 65 ± 10.9 Gender: 11m/5f H&Y: 1–4 HOA (*n* = 17) Age: 74.4 ± 5.8 Gender: 9m/8f	ON	Overground 6 m walkway.	Ten trials at preferred walking speed. Three or more consecutive cycles recorded.	Lower distal muscle activity (particularly TA during single support) and greater proximal muscle activity (swing phase) in PD vs. HOA. Difference in distal muscles between PD and CA; lower activity of TA and gastrocnemius (early stance). In PD group, there was reduced ∆EMG/∆ankle angle for the plantarflexors compared to HOA.
Rodriguez et al.^70^	Investigated differences in motor modules between individuals with PD and HOA. Compared muscle weighting vectors and activation profiles of the motor modules between individuals with PD and HOA. Relationships between motor modules and gait mechanics in PD and HOA.	PD (*n* = 15) Age: 66.6 ± 7.8 Gender: not reported HOA (*n* = 14) Age: 66.2 ± 7.1 Gender: not reported	ON	Split-belt treadmill	Ten minutes at self-selected walking speed.	PD required fewer modules compared to healthy controls. Descriptively, PD exhibit an altered temporal activation profile of modules. The percent variance accounted for MG, SM and BF was significantly higher for PD. No significant difference in speed between PD and HOA.
Rose et al.^72^	Investigated the effect of 8-week high intensity locomotor training using a positive-pressure treadmill on knee extensor flexor and extensor activity.	PD (*n* = 13) Age: 62 ± 6.4 Gender: 13m H&Y: 2–3 HOA (*n* = 8) Age: 53 ± 4.4 Gender: 8m	ON	Anti-gravity treadmill	Five walking trials 70 s long at 3 km/h treadmill speed. Three recordings taken: Training day 2 Midway through training Post training Eight-week treadmill training (1 h × 3/week) involving running, walking, bodyweight support, different speeds and varied locomotion (chassé, skipping, jumping, sprints)	Knee extensors VL and VM were active for a longer proportion of the gait cycle and displayed higher peak activation in PD vs. HOA. BWS decreased activity duration of knee extensors but increased knee flexor duration.

*BWS* body weight support, *CA* cerebellar ataxia, *FOG* freezing of gait, *FW* fast walking, *GC* gait cycle, *HOA* Healthy Older Adults, *HYA* healthy young adults, *H&Y* Hoehn and Yahr, *PD* Parkinson’s disease, *RAS* rhythmic auditory stimulation, *RQA* recurrence quantification analysis, *STN-DBS* subthalamic nuclei deep brain stimulation, *UPDRS-III* Movement Disorders Society Unified Parkinson’s Disease Rating Scale III, *VAF* variance accounted for, *Muscles – BF* biceps femoris, *GM* gluteus medius, *LG* lateral gastrocnemius, *MG* medial gastrocnemius, *SM* semi-membranosus, *RF* rectus femoris, *TA* tibialis anterior, *TS* triceps surae, *VL* vastus lateralis, *VM* vastus medialis.

**Table 3:** Aims, protocol and key findings of intervention studies.

Study	Aims	Participant characteristics	Medication	Walking surface	Walking task	Key findings
Caliandro et al.^71^	Evaluated TA EMG in the ‘OFF’ and ‘ON’ during late swing/early stance. Examined relationship between TA activity and UPDRS-III.	PD (*n* = 30) Age: 70.7 ± 5.5 Gender: 12m/18f UPDRS-III: r-TA 4.5 ‘ON’ 19.5 ‘OFF’ UPDRS-III: nr-TA 5.0 ‘ON’ 8.5 ‘OFF’*	ON OFF	Overground 10 m walkway.	Minimum of two trials of walking at self-selected speed. Assessed on separate days for ‘ON and ‘OFF’ states.	9/30 participants showed less TA activity (late swing–early stance) in ‘OFF’ vs. ‘ON’ state (r-TA subgroup). These individuals also had lower UPDRS-III following L-DOPA and increased walking speed. Remaining participants (nr-TA subgroup) exhibited no change in TA activity between ON and OFF state (nr-TA subgroup).
Cioni et al.^64^	Investigated EMG of the lower limb during walking in the ‘OFF ‘and ‘ON’ medication states in PD and compared with HOA.	PD (*n* = 15) Age: 58.3 ± 13.5 Gender: 13m/2f H&Y: 1–4 HOA (*n* = 10), Age: 58 ± 7.6 Gender: 13m/2f	ON OFF	Overground 8 m walkway	Walked at self-selected walking speed for at least ten gait cycles.	OFF*:* TA reduced activity. Decreased activity of ankle plantarflexors (late stance). Prolonged activity of knee extensors (stance). ON*:* TA (early stance/late swing) and TS (late stance) increased. TA increase correlated with increased cadence. Hip and knee joints were more flexed vs. HOA (stance) and correlated with hamstring activation.
Ferrarin et al.^68^	Analysed effects of unilateral and bilateral subthalamic nucleus stimulation on lower limb EMG during walking in individuals with PD.	PD (*n* = 10) Age: 60.2 ± 4.8 Gender: not reported H&Y: 3.7 ± 0.7 UPDRS-III: ~21 HOA (*n* = 10) Age: 59.2 ± 4.5 Gender: not reported	OFF	Overground 10 m path	Walked at preferred walking speed and completed four conditions: Stimulation off Stimulation on (bilat STN) Stimulation on (right STN) Stimulation on (left STN)	‘OFF’ state vs. bilateral stimulation: longer activation of SM and RF during stance. MG and TA showed reduced activity at push-off and also at initial stance for TA. ‘OFF’ state vs. unilateral STN: increased activity in distal muscles only; TA (double stance) and MG (late single stance).
Pourmoghaddam et al.^60^	Investigated if an index derived from multiple muscle RQA analysis could detect changes in walking speeds and levodopa intake during treadmill walking.	PD (*n* = 9), Age: 76 ± 6 Gender: 9m H&Y: 2–3 (ON) UPDRS-III: 28.6 ± 4.6 (ON)	ON OFF	Treadmill	Two minutes at a self-selected walking speed. Treadmill speed increased by 0.045 ms^−1^ every five strides up to a max of 180 s both ON and OFF.	In ‘ON’ state, index significantly reduced but increased with gait speed. No significant interaction between gait speed and medication. The researchers considered the index to be a measure of multi-muscle activation. They concluded collective overall activity of muscles was decreased in the ON state compared to OFF.
Rizzone et al.^54^	In people with PD, bilaterally implanted for STN-DBS investigated: If a subgroup with a dominant STN were present. Effect of unilateral DBS of the dominant STN on EMG during walking and effect on UPDRS score.	PD (*n* = 10) Age: 60.2 ± 4.8 Gender: 5m/5f H&Y: 3.7 ± 0.7 (ON) UPDRS-III: 59.9 (OFF) 21.2 (ON) HOA (*n* = 10) Age: 61.4 ± 5.0 Gender: 5m/5f	OFF	Overground 10 m path	Walking at self-selected speed for four conditions: Stimulation off Stimulation on (bilat STN) Stimulation on (right STN) Stimulation on (left STN)	Six participants were identified with a ‘dominant STN’. Dominant STN stimulation and bilateral stimulation increased activity of TA (first double support), MG (push off), RF (first double support) and SM (late swing), alongside Improved motor UPDRS score, but not UPDRS gait score. Bilateral stimulation of the STN increased TA activity (first double stance) and MG (push off).
Roemmich et al.^61^	Investigated effects of dopaminergic therapy on number, structure and timing of motor modules. Assessed the relationship between motor modules and speed in individuals with PD.	PD (*n* = 9) Age: 65.7 ± 7.3 Gender: 7f/2m UPDRS: 41 ± 10 (OFF), 37 ± 7 (ON)	OFF ON	Overground walkway Split belt treadmill	Ten overground gait trials at self-selected pace, treadmill at preferred walking speed for 5 min whilst holding on handrails and wearing harness.	No significant differences were found in the number, structure and timing of motor modules between the ON and OFF state of patients. A lower %VAF was found for treadmill walking compared to treadmill walking in OFF.

*BWS* body weight support, *CA* cerebellar ataxia, *FOG* freezing of gait, *FW* fast walking, *GC* gait cycle, *HOA* Healthy Older Adults, *HYA* healthy young adults, *H&Y* Hoehn and Yahr, *PD* Parkinson’s disease, *RAS* rhythmic auditory stimulation, *RQA* recurrence quantification analysis, *STN-DBS* subthalamic nuclei deep brain stimulation, *UPDRS-III* Movement Disorders Society Unified Parkinson’s Disease Rating Scale III*, VAF* variance accounted for, *Muscles* — *BF* biceps femoris, *GM* gluteus medius, *LG* lateral gastrocnemius, *MG* medial gastrocnemius, *SM* semi-membranosus, *RF* rectus femoris, *TA* tibialis anterior, *TS* triceps surae, *VL* vastus lateralis, *VM* vastus medialis.

Parameters derived from EMG signals included amplitude related measures^54,58,62–66,68,70,71,73^, duration of activity^72^, coactivation indices^63,67,73^, multi-muscle activation^61,70^, variability^60,69^ and symmetry^69^. Amplitude normalisation was applied to the peak value obtained during walking in four studies^61,67,70,73^ and to the average amplitude in five studies^58,59,63,69,71^. One study normalised to the 95th percentile of the control group^54^ and another to maximum isometric voluntary contractions^72^. Four studies did not report amplitude normalisation methods^54,62,64,68^.

The number of gait cycles included for analysis ranged from ten^58,59,64,65^ to a minimum of twenty^58,59,63,66^. Some studies only described the number of trials^54,71^ or walking time duration^70^. Several did not specify the number of cycles^60,62,68,69^. EMG parameters were evaluated for different phases such as the entire gait cycle, initial/mid/terminal stance and early/late swing.

### Muscle activity

Six studies compared differences in lower limb EMG activity patterns during walking between individuals with PD in the ON state and HOA^59,62,64–66,72^. Five studies reported conflicting findings regarding TA activity during walking. Three studies reported a reduction in MG amplitude^63,65,66^ during stance in individuals with PD. Three studies found differences in proximal muscle activity in the ON state with greater and more prolonged activity of proximal lower limb muscles in people with PD compared to HOA^64,65,72^. Two studies investigated differences in variability of lower limb muscle activity^58,69^. BF, TA and MG displayed greater variability in amplitude in individuals with PD compared to HOA, although MG displayed lower timing variability^58,69^. Three studies assessed multi-muscle activity through analysis of coactivation^63,67^ or muscle synergies^70^.

Four studies compared muscle activity during walking in the ON state with activity during the OFF state^60,61,64,71^. Two studies recorded increased TA activity during late swing/early stance in the ON state^64,71^. Cioni et al.^64^ additionally observed increased activity of plantarflexors during late stance^64^. A decrease in multi-muscle regularity derived from recurrent quantification analysis during the ON state was reported by Pourmoghaddam et al.^60^. Roemmich et al.^61^ observed that composition of muscle synergies, not the number of synergies accounting for 95% of variance, differed between the ON and OFF states, with the synergies to which VM and RF had higher weightings accounting for a greater amount of variance in the OFF state compared to the ON state^61^.

Two studies investigated the effect of DBS on muscle activity, with both applying DBS to the subthalamic nuclei (STN). The TA, MG, SM and RF muscles were reported to increase activation following DBS^54,68^.

Caliandro et al.^71^ described that individuals with PD who displayed a reduction in TA activity during initial stance in the OFF state had better motor function (decreased Movement Disorders Society-Unified Parkinson’s Disease Rating Scale III (MDS-UPDRS-III)) in the ON state, compared to individuals who demonstrated no difference in TA activity between ON and OFF^71^. Arias et al.^67^ reported no relationship between muscle coactivation and gait kinematics^67^.

## Discussion

To the best of our knowledge, this is the first systematic review to report on EMG in individuals with PD during walking and the effect of dopaminergic therapy and DBS on motor behaviour. Of the sixteen studies identified, the majority reported differences in EMG parameters such as the timing and amplitude of muscle signals and muscle synergies between individuals with PD and HOA. However, in many cases results were conflicting due in part to differing protocols. Only six studies investigated the effect of dopaminergic medication or DBS on EMG activity. Notably, most studies did not relate EMG to gait or clinical measures evaluating motor symptoms severity such as the MDS-UPDRS III, indicating a major limitation in functional interpretation of EMG features and understanding gait in PD. The analysis of EMG signals in isolation without gait kinematics and kinetics or clinical measures restricts its application. Understanding the relationship between muscle activity and gait features will help identify which muscles and activation patterns underpin gait impairment and provide evidence-based support for improving the effectiveness of rehabilitation interventions by targeting specific muscles and muscle groups.

### How does PD affect muscle activity?

Although differences were detected between individuals with PD and HOA, there was limited consensus regarding findings, particularly for TA, the most frequently assessed muscle. Cioni et al.^64^ reported that the TA displayed similar activity patterns in individuals with PD and HOA^64^. By contrast, Dietz et al.^59^ observed greater TA activity and Mitoma et al.^65^ reported less activity in individuals with PD^59,65^. Jenkins et al.^62^ found TA peaked later in PD compared to healthy adults^62^. Albani et al.^66^ recorded differences in TA in the ON state between freezers and non-freezers with greater activity bilaterally in the swing phase in freezers compared to HOA, whilst non-freezers showed greater activity only in the left TA^66^. These findings suggest differences in motor control of gait between people who exhibit freezing of gait (FoG) and those who do not freeze. The contradictory findings for TA may be accounted for in part by different processing methods and protocols. Dietz et al.^59^ and Mitoma et al.^65^, for example, did not normalise the amplitude of the signals^59,65^ which precludes comparison of EMG amplitudes between groups (Tables 4, 5). The walking studies were conducted on different surfaces including a level overground walkway and motorised treadmill. Warlop et al.^74^ reported that treadmill walking differed in individuals with PD compared to overground walking, therefore direct comparison between EMG signals collected on different surfaces may give misleading results^74^. Another reason for differences in TA activity is the heterogynous nature of PD, with differences in phenotype (tremor-dominant and postural instability and gait disturbance), disease duration, symptom severity and features such as FoG. Functionally, decreased TA activity reduces foot clearance^75^ and alters foot contact patterns which may influence fall risk. A shorter duration of TA muscle activity occurs prematurely in individuals with PD prior to freezing^76^.

**Table 4:** Methodology and signal processing techniques for non-intervention studies.

Study	Lower limb muscles Electrode placement	EMG Signal Processing	EMG outcome measure	Gait parameters reported	Gait duration analysed
Albani et al.^66^	Bilateral	Rectified	RMS	Temporal/spatial	Final 20 GCs per trial
	Distal:	No details of filter or method calculating RMS reported.		Walking speed	
	TA, MG				
	Electrode placement:	Normalisation			
	Not specified	Not reported.			
Arias et al.^67^	Bilateral	Rectified	Coactivation index	Temporal/Spatial	Minimum of 16 GCs per subject
	Distal:	Bandpass: 20–450 Hz		Walking speed	
	TA, SO	Low-pass: 10 Hz		Cadence	
	Electrode placement: According to Cram et al. 1998	Normalisation		Step length	
		(a) No time normalisation reported.			
		(b) Amplitude normalised to peak baseline gait values.			
Bello et al.^73^	Unilateral;	Bandpass: 10–500 Hz	RMS	Temporal/spatial	Average of 3 trials
	most affected leg (PD)	RMS: 50 ms window	Coactivation ratio of antagonistics at ankle and knee joints (VL-BF and TA-MG) per gait phase.	Walking speed	Third minute of treadmill walking
	right leg (HOA)	Normalisation			
	Proximal:	(a) Time normalised to GC and divided into load, single, pre-swing and swing gait phases.			
	VL, BF	(b) Amplitude normalised to peak value during overground walking			
	Distal:				
	TA, MG				
	Electrode placement: SENIAM				
Dietz et al.^63^	Bilateral	Rectified	iEMG	Temporal/Spatial	20 GCs
	Distal:	Bandpass: 3–1000 Hz	Co-activity index	Stance time	
	TA, MG	iEMG calculated for 1/20th s of GC.		Swing time	
	Electrode placement:	Normalisation		Stance length	
	Not specified	(a) Time normalised to % of GC.		Stride frequency	
		(b) Amplitude normalised to walking at 0.75 m/s		Kinematic	
				Knee and ankle joint angles	
Dietz et al.^58,59^	Bilateral	Rectified	RMS	Kinematic	Minimum of 10 GCs
	Proximal:	Bandpass: 30–300 Hz		Ankle and knee joint angles	
	BF, RF	Averaged over 20 GCs.			
	Distal:	RMS determined for entire GC.			
	TA, MG	Normalisation			
	Electrode placement:	(a) Time normalised to % of GC.			
	Not specified	(b) Amplitude normalised to normal body loading.			
	Electrode placement				
Jenkins et al.^62^	Bilateral	Rectified	iEMG	Temporal/spatial	Five walking trials over instrumented mat per condition
	Proximal:	Low-pass: 6 Hz	Time to peak activity	Walking speed	
	Quadriceps, BF	EMG analysed for 4 phases: initial stance, midstance, terminal stance, swing		Step Length	
	Distal:	Normalisation		Step length variability	
	TA, LG	(a) Time normalised to 100% of GC.		Single-limb support	
	Electrode placement:	(b) No amplitude normalisation reported.			
	Not specified				
Miller et al.^69^	Bilateral;	Rectified.	Ensemble average, variability, symmetry	Temporal/spatial	
	Proximal	Bandpass: 30–250 Hz		Stance,Step length	
	VL	Low-pass: 10 Hz		Speed	
	Distal	Ensemble average over 6 GCs calculated.		Double/single support phase	
	TA, MG	Normalisation		Kinematic	
	Electrode placement: Not specified	(a) Time normalised to 128 point GC.		Hip, knee and ankle joint angles	
		(b) Amplitude normalised to unit intensity.		Kinetic	
				Ground reaction force	
				CoP Displacements	
Mitoma et al.^65^	Bilateral	Rectified	iEMG	Temporal/spatial	10 GCs
	Proximal:	Bandpass: 20–500 Hz	Change in EMG	Stance,Step length	
	AM, GM, VL, BF	Integrated: 50 ms	between gait phases.	Speed	
	Distal:	Data were split into four phases: 1st double support, single support, 2nd double support, swing.	Ratio of EMG change to joint angle change.	Double, single support phase	
	TA, gastrocnemius, SO	Normalisation		Kinematic	
	Electrode placement:	Not reported.		Hip, knee, ankle joint angles	
	According to Knuttson & Richards (1979)			Kinetic	
				Ground reaction force	
				Centre of pressure.	
Rodriguez et al.^70^	Bilateral	Demeaned, Rectified	Motor modules	Temporal/spatial	Last 4 min of treadmill walking
	Proximal:	High-pass: 35 Hz		Walking speed	
	GM, RF, VM, ST, BF	Low-pass—7 Hz		Kinetic	
	Distal:	Nonnegative matrix factorisation applied.		Sagittal hip/ knee/ankle moment impulses	
	TA, MG, SO	Normalisation			
	Electrode placement:	(a) Time normalised to 100% of GC.			
	Not specified	(b) Amplitude normalised to peak trial values.			
Rose et al.^72^	Bilateral	RMS: 21 ms window	RMS: 21 ms window	Kinetic	20 s of walking
	Proximal:	Normalisation	Normalisation	Knee joint torque	
	VL, VM, SM, BF	(a) No time normalisation reported.	(a) No time normalisation reported.	Ground reaction force	
	Electrode placement: According to Perotto et al. 2005.	(b) Amplitude normalised to maximum value during isometric maximum voluntary contractions	(b) Amplitude normalised to maximum value during isometric maximum voluntary contractions		

*CC* correlation coefficient, *COV* coefficient of variation, *DC* direct current, *GC* Gait cycle, *HOA* healthy older adult, *iEMG* integrated EMG, *RMS* root mean square, *ROM* range of motion, *RQA* recurrence quantification analysis, *SENIAM*, surface EMG for non-invasive assessment of muscles, *SR* sampling rate, *%DET* percent determinism. **Muscle:** *AM* adductor magnus, *BF* biceps femoris, *GM* gluteus medius, *LG* lateral gastrocnemius, *MG* medial gastrocnemius, *PL* *peroneus longus*, *SM* semimembranosus, *ST* semitendinosus RA, *RF* rectus femoris, *TA* tibialis anterior, *TFL* tensor fascia latae, *VL* vastus lateralis, *VM* vastus medialis.

**Table 5:** Methodology and signal processing techniques for intervention studies.

Study	Lower limb muscles Electrode placement	EMG Signal Processing	EMG outcome measure	Gait parameters reported	Gait duration analysed
Caliandro et al.^71^	Bilateral	Rectified	Peak RMS for TA at	Temporal/spatial	Two trials of 10 m for each session
	Distal:	High-pass: 50 Hz	Late swing to early stance	Walking speed	
	TA, MG	Low-pass: 7.5 Hz			
	Electrode placement: According to Rainoldi et al. 2004	RMS: 50 ms window			
		Normalisation			
		Not reported.			
Cioni et al.^64^	Bilateral	Rectified	iEMG	Temporal/Spatial	Minimum of 10 GCs
	Proximal:	Time averaged at 50 Hz		Walking speed	
	Quadriceps, hamstrings			Stance (% GC)	
	Distal:	Normalisation		Stride length	
	TA, triceps surae	(a) Time normalised to 100% of GC in 2% increments.		Cadence	
	Electrode placement:	(b) No amplitude normalisation reported.		Kinematic	
	Not specified			hip, knee, ankle joint angles	
Ferrarin et al.^68^	Bilateral;	Rectified.	RMS	Temporal/Spatial	
	Proximal	Bandpass: 10–200 Hz		Speed	
	RF, SM	High-pass: 50 Hz		Stride length	
	Distal	Low-pass: 7 Hz		Cadence	
	TA, MG	RMS calculated for: 1st double support, early single support, late single support, 2nd double support, early swing, late swing.		Velocity	
	Electrode placement: SENIAM	Normalisation		Stance	
		(a) Time normalised to 100% of GC.		Kinematic	
		(b) No amplitude normalisation reported.		Hip, knee and ankle ROM	
				Kinetic	
				Peak joint moments and powers at the hip, knee and ankle	
Pourmoghaddam et al.^60^	Unilateral: right leg	Bandpass: 20–460 Hz.	Index based on algorithm composed of products of %DET.	Temporal/Spatial	Up to 180 s overground walking before and after taking medication.
	Proximal:	RQA applied. %DET calculated for individual muscles.		Walking speed	
	RF, VM, BF	‘Synergos’ index determined using algorithm involving products of %DET.			
	Distal:	Normalisation			
	TA, LG, SO	None reported			
	Electrode placement:				
	Not specified				
Roemmich et al.^61^	Bilateral;	Demeaned, rectified.	Motor modules	Temporal/Spatial	10–20 GCs before and after medication
	Proximal	High-pass: 35 Hz		Velocity	
	GM, RF, VM, SM, BF	Low-pass: 7 Hz		Kinematic	
	Distal	Nonnegative matrix factorisation applied.		Stride length	
	TA, MG, SO	Normalisation		Step length	
	Electrode placement: Not specified	(a) Time normalised to 100% of GC.		Stride time	
		(b) Amplitude normalised to peak trial values.		Step time	
Rizzone et al.^54^	Bilateral	Rectified	RMS	Temporal/Spatial	Eight trials in each condition
	Proximal:	Bandpass: 10–200 Hz.		Speed,Stride length	
	RF, SM	High-pass: 50 Hz		Cadence	
	Distal:	Low-pass: 7.5 Hz		Stance time	
	TA, MG	RMS calculated for 4t gait phases:		Kinematic	
	Electrode placement:	Normalisation		Hip, knee, ankle ROM	
	Not specified	(a) Time normalised as % of stride duration.		Kinetic	
		(b) No amplitude normalisation reported.		Hip, knee and ankle joint power and moments	

*CC* correlation coefficient, *COV* coefficient of variation, *DC* direct current, *GC* gait cycle, *HOA* healthy older adult, *iEMG* integrated EMG, *RMS* root mean square, *ROM* range of motion, *RQA* recurrence quantification analysis, *SENIAM* surface EMG for non-invasive assessment of muscles, *SR* sampling rate, *%DET* percent determinism. **Muscle:** *AM* adductor magnus, *BF* biceps femoris, *GM* gluteus medius, *LG* lateral gastrocnemius, *MG* medial gastrocnemius, *PL* peroneus longus, *SM* semimembranosus, *ST* semitendinosus RA, *RF* rectus femoris, *TA* tibialis anterior, *TFL* tensor fascia latae, *VL* vastus lateralis, *VM* vastus medialis.

Studies investigating the activity of MG muscle in individuals provided more conclusive results, with the majority reporting reduced activity in the PD group compared to HOA. As the MG muscle is important for forward propulsion of the body and vertical support^77^, a decrease in activity may result in reduced gait speed and loss of postural balance along the vertical axis. Three studies reported prolonged increased activity of knee flexors and extensors^64,65,72^ in individuals with PD. Biomechanically, the enhanced proximal muscle activity may compensate for the reduced function of distal muscles. Greater contraction of the quadriceps during the stance phase will increase extension of the knee, leading to greater stability in this joint during single stance which may compensate for reduced stability at the ankle joint. Greater activity of hamstrings during swing will increase hip extension and knee flexion and may replace some of the foot placement and initial loading role of the distal muscles acting on the ankle joint. Increased muscle activity entails a larger metabolic demand which may limit walking speed and mobility^78^. Differential compensatory changes in lower limb muscles during walking have been observed in other neurological pathologies such as post-polio syndrome and stroke^79,80^.

Other EMG measures determined in the reviewed articles included variability, coactivation, muscle synergies and asymmetry. Two studies assessed variability of EMG amplitude and reported greater variability of EMG for proximal and distal muscles^58,69^. Increased EMG variability suggests decreased automaticity of locomotor control in PD resulting from the dysfunctional putamen^81^. Clinically, greater gait variability is associated with higher falls risk in individuals with PD and HOA^33^. However, the relationship between variability and stability is complicated with a certain level of variability essential to enable adaption to perturbations^82^. There was conflicting evidence regarding changes in coactivation of agonists and antagonists in lower limb muscle pairs during walking in individuals with PD. Dietz et al.^63^ observed increased coactivation of TA and MG in people with PD during treadmill walking compared to HOA whereas Arias et al.^67^ reported no difference in coactivation of TA and SO when walking overground^63,67^. A motorised treadmill can act as an external cue resulting in reduced gait variability and altered coordination of muscles^74^. Only one study assessed muscle synergies and observed fewer muscle synergies accounting for 95% of variance, altered temporal profiles and a higher percentage of variability accounted for by MG, SM and BF in the PD group compared to HOA^70^. A reduction in muscle synergies suggests a simpler, possibly less robust, control system^83^. Miller et al.^69^ reported higher asymmetry in TA and MG activity in PD compared to HOA^69^. Motor and gait asymmetry are early features of PD^84–86^. Greater asymmetry is associated with the reduced integrity of callosal sensorimotor regions^87^ and impairment in sensorimotor integration, in addition to an increased risk of falls^88^.

### How is muscle activity modified by interventions?

Altered contraction of individual muscles and coordination of activity between muscles underpin gait impairment in PD. Interventions targeting gait dysfunction must therefore modify activity of individual muscles and activation patterns. Evidence from the reviewed studies indicate that dopaminergic medication^64,71^ and STN-DBS^54,68^ increase the activity of distal lower limb muscles, particularly of the TA muscle. The TA has been reported to have greater projections from the cortex to its motoneurons compared to other lower limb muscles which may account for this muscle being targeted more^89^. The effect of enhanced muscle contraction, providing there is no increase in the antagonist muscle, is to increase the forces acting about a joint (joint moments). The functional consequence of this is increased angular velocity resulting in increased gait velocity which has been observed to occur following dopaminergic medication and STN-DBS, achieved mainly through longer step length^90,91^. In individuals with PD, the plantarflexors are impacted more than the dorsiflexors and there is no evidence that the dorsiflexors are weaker in individuals with PD compared to HOA. Increasing disproportionately the activity of the dorsiflexors relative to the plantarflexors will produce an imbalance in forces around the ankle joint with possible associated instability. The effect of STN-DBS on muscle function differs from that of dopaminergic medication as it increases activity of both proximal and distal lower limb muscles. Individuals with PD generally exhibit decreased activity of distal muscles and greater activity of proximal lower limb muscles as outlined in the previous section. A further increase in proximal lower limb muscle due to STN-DBS, may result in imbalance of forces across and between joints and contribute to aggravation of FoG and postural instability which has been reported following STN-DBS^92^.

Only one study assessed variability of gait EMG following dopaminergic medication. Pourmoghaddam et al.^60^ observed decreased multi-muscle regularity, determined through nonlinear analysis methods, during the ON state^60^. This implies increased variability of EMG patterns which could contribute to postural stability not being well controlled by dopaminergic medication, although more evidence is needed in support^91^. Two studies have reported that step time variability decreased with dopaminergic medication and Gilat et al.^93^ observed this variability was associated with altered striatal, limbic and cerebellar activity^93,94^.

Dopaminergic medication and STN-DBS modulate activity of similar brain structures and networks with some differences reported. Evidence indicates that dopaminergic medication and STN-DBS suppress the primary motor cortex (M1)-STN beta band (13–35 Hz) coherence^95–98^. Studies investigating cyclical movements of upper and lower limbs have found cortico-muscular beta coherence to be enhanced following dopaminergic medication^99,100^. STN-DBS has similarly been observed to increase cortico-muscular beta coherence in hand tremor^101^. Increased cortico-muscular beta band coherence has been linked with greater muscle activity^102^. Mueller et al.^103^ additionally reported dopaminergic medication increased connectiveness between the putamen and both the cerebellum and brainstem, with high connectivity correlated with a better motor score (UPDRS-III)^103^. STN-DBS has also been found to increase activity of motor cortical regions during movement and decrease activity during rest, with lower cortical activity during rest associated with clinical improvement^98^. These differences in brain targets may account for the varying effects dopaminergic medication and STN-DBS have on gait. In postural studies, dissimilar outcomes have also been reported, with dopaminergic medication increasing postural sway area whereas STN-DBS reduced postural sway area^104^.

### What is the quality of the reviewed studies?

Overall, quality scores were mediocre for both non-intervention and intervention studies. The main points that studies scored low on were sample size justification, electrode placement procedures and signal processing techniques. Individuals with PD exhibit great heterogeneity and generally high inter- and intra- subject gait EMG variability^105^ necessitating greater sample sizes than for HOA. However, the median sample size was only twenty-two and no study in this review performed power analysis to justify their selection of participant number. Most studies included a greater proportion of males, reflecting the gender bias in PD although some studies did not specify gender. Gender differences in muscle activity during walking have previously been reported^106,107^ indicating it is an important factor. Only four studies determined electrode location using validated guidelines such as the SENIAM guidelines^108^. Identification of the optimal electrode site helps ensure the signals with higher signal to noise ratio are recorded from the selected muscle with minimal cross-talk from adjacent muscles^109^.

Over half of the studies did not report any signal normalisation methods^59–61,63,65,66,69–71^. Such normalisation is essential to allow comparisons of EMG between muscles, sessions and participants as factors such as thickness of adipose tissue, presence of oedema and number and orientation of muscle fibres will modify amplitude^110,111^. Excluding normalisation can invalidate subsequent results.

For the intervention specific studies, all studies excluded reports of adverse events and two studies did not state whether the researchers were blinded from measuring the main outcomes^54,68^. Reporting of adverse events is crucial for ensuring participant safety and determining potential confounding factors which may influence results interpretation and subsequent intervention development.

### Limitations of reviewed studies

A small selection of superficial lower limb muscles was assessed during walking in individuals with PD with certain muscle groups studied less. Information about the contribution of muscles to movement is necessary for understanding compensatory mechanisms resulting in impaired gait and dynamic postural control and for developing interventions. Only one study recorded the hip adductors, a muscle group with a cross-sectional area (CSA), which relates to muscle force, comparable to the CSA of the quadriceps group, and almost three times greater than the CSA of the hamstrings^112^. This creates a vacuum in our knowledge of motor activity during walking in PD particularly given that mediolateral sway and instability are greater in individuals with PD^113^. The reviewed studies reported group differences in a wide range of EMG parameters including temporal information (muscle onset/offset), amplitude (root mean square, integrated EMG, mean amplitude of EMG), coactivation indices, synergies, symmetry/variability indices and nonlinear indices. However, spectral characteristics of the EMG signals and intermuscular coherence, which may provide information about motor unit recruitment and neuronal networks controlling muscle activity, were not analysed.

All studies were conducted in a gait laboratory with participants being closely observed whilst walking under constrained conditions. Spatio-temporal measures of gait and by implication muscle activity are modified when gait is observed overtly rather than covertly^114^. Only single-task walking was generally assessed. However, real-world walking involves additional activities such as walking and turning, varying walking speeds, completing complex visuomotor tasks and talking^115,116^. As an individual’s EMG profile will vary from day to day^117^, recording over multiple days and over a longer time period could permit a more accurate appraisal of motor activity to be made and also determine how motor activity changes over time and with disease progression. Repeat measurements are particularly important for individuals with PD as they will exhibit considerable fluctuation in gait depending on their medication regimes.

Thus, the current information regarding EMG activity during gait in PD is restricted in its ability to reflect the complexity of real-life walking and the capacity of the nervous system to integrate multiple neural networks to ensure safe efficient walking and facilitate gait adaptations in response to varying environmental demands. Measurement of muscle activity patterns during real-world gait over longer time periods would capture the specific motor control strategies used under these conditions that would otherwise be confounded by testing in a controlled environment. There are, however, challenges with monitoring free-living EMG, given the high sampling rate needed and low signal to noise ratio compared to wearable sensors such as accelerometers.

### Limitations

This systematic review carries the usual limitations regarding restrictions imposed by the nature of literature selection. Only English-language journals were included. Studies involving gait initiation, freezing episodes, running and upper limb muscles/ tasks were excluded as inclusion criteria stipulated only walking tasks. Further studies are required to understand task-based differences in EMG activity between PD and controls as at present there is insufficient evidence in the literature to conduct this type of review.

### Recommendations

This review has raised many issues regarding the limitations surrounding our current knowledge of motor activity during walking in individuals with PD. Recommendations for future studies are provided below and divided into points relating to study protocol and data processing.

### Protocol considerations for EMG

- Real-world walking. Investigating gait during real-world activity is desirable to understand motor strategies in a natural environment although current technological limitations make long term recordings challenging.
- Sample size. Greater numbers of participants and more stride cycles are necessary.
- Muscle selection. Muscles representing all major muscle groups acting on the ankle, knee and hip joints in the sagittal and coronal planes should ideally be recorded to permit analyses of multi-muscle activation patterns and underlying neural control systems to be undertaken.
- Electrode placement. A clear statement must be included regarding methods used to identify electrode placement and established guidelines followed.
- Longitudinal studies. This will inform us how motor patterns change with age and disease progression and help establish EMG characteristics as biomarkers.
- Additional gait and cortical parameters. Parameters such as joint kinematics and kinetics as well as cortical activity measured with mobile, wireless systems such as functional near infrared spectroscopy or electroencephalography will enable us to relate EMG to gait impairment and cortical processes.

### Data analytical considerations for EMG

- Filtering and normalisation. Appropriate filtering techniques must be performed to ensure signals are physiological and not convoluted by noise. Normalisation techniques must be applied to each muscle for each individual to allow comparisons.
- Parameter selection. Parameters should be selected that reflect underlying neural control systems, physiology and gait dysfunction. Spectral analysis, nonlinear analysis of variability, and factor analysis methods, such as nonnegative matrix factorisation, may indicate neurophysiological mechanisms. Relating EMG outcome to specific gait functions such as loading, push-off and swing is important for identifying targets for gait rehabilitation in PD.

## Conclusion

Results from this review indicate individuals with PD have decreased activity of distal lower limb muscles, specifically plantarflexors, and increased activity of proximal lower limb muscles during walking compared to HOA. Variability of EMG of lower limb muscles during walking is increased in individuals with PD. Dopaminergic medication enhances activity of distal muscles and STN-DBS increases both proximal and distal muscle activity during walking. The effect of further increase in proximal muscle contraction may contribute to FoG and gait instability associated with STN-DBS. There is insufficient evidence to state how changes in muscle activation patterns directly relate to altered temporospatial gait parameters.

The findings from this review highlight the paucity of information regarding how muscles contract during walking in people with PD and how this activity relates to gait impairment. This lack of information about muscle activity is in marked contrast to the wealth of knowledge we have concerning spatio-temporal features of gait or neurodegenerative changes in the brain, requiring invasive techniques. Consequently, although gait impairment is common in PD, we cannot identify which muscles are responsible for slower walking speed or shorter steps, or why falls occur more commonly.

It is not feasible, due to insufficient data, to differentiate individuals with PD from HOA through analysis of muscle activity. Further studies must be undertaken, to enable gait EMG to be employed as a biomarker of PD and to generate personalised rehabilitation techniques targeting dysfunctional muscles. The future challenge is to develop a multi-centre project involving a large cohort of individuals with PD and HOA, which investigates a comprehensive set of muscles and extracts a range of parameters from the EMG over an extended time-period in different settings.

## Methods

### Search strategy

A literature search was performed in December 2019 to identify relevant articles in the following databases by one author (AI): MEDLINE (1946–2019), Embase (1974–2019), Scopus and Web of Knowledge (Table 6). The search extended back to 1946 to include articles published in the 1960s when surface EMG was first introduced, and patients were first prescribed levodopa.

**Table 6:** Search fields with their corresponding search term used.

Measurement technique	Population	Gait	Data analysis
Electromyography	Parkinson’s Disease	Walk*	Muscle synerg*
Surface EMG		Gait	Muscle activit*
Invasive EMG		Stance	Muscle patterns
		Step*	Coherence
		Stride	Coactivation
		Swing	Cocontraction
		Speed	
		‘Double limb’	
		Dorsiflex*	
		Plantarflex*	
		Locomot*	
		Ambul*	
		Pace	
		Rhythm	
		Tread	
		Asymmetr*	
		Symmetr*	
		Variability	
		Frequency	
		Velocity	

Four search fields were selected linked with the conjunction ‘’OR”. MESH headings were used for Medline and Embase. Synonyms for each key term were applied. The first search field comprised the measurement technique of interest (EMG) with surface and wire/needle EMG included. The second search field focused on Parkinson’s only, excluding atypical PD and other parkinsonian disorders. The third field consisted of synonyms for walking tasks and gait characteristics. The final search field contained descriptors for the data analysis used (e.g. muscle activation patterns, muscle synergies). The searches from all four databases were combined into a citation manager with duplicates removed. Three authors (AI, AP, LA) screened suitable titles and abstracts. Full text review was performed if the suitability of a paper for inclusion was unclear. Reference lists were manually scanned during full text review to identify relevant articles.

### Inclusion and exclusion criteria

Articles recording the EMG signal in individuals with PD during forward, straight line walking were included. Studies which focused on specific phases of walking such as turning, gait initiation and termination or a special type of walk such as backward walking or walking in the Timed Up and Go (TUG) test were excluded. Studies that only analysed static standing, posture and tremor or specific gait events observed in PD such as freezing of gait were excluded. Studies involving groups with pathologies outside of PD were excluded. Dopaminergic studies (ON/OFF) and DBS studies were only included when the EMG signal during a walking task was reported. Only articles written in English were considered. Reviews, abstracts, cohort studies, case studies, editorials, commentaries, discussion papers, conference proceedings and studies lacking full text were excluded. Eligibility and inclusion were determined by three reviewers (AI, AP, LA). Discrepancies were resolved through discussion resulting in a unanimous decision or a majority consensus.

### Data extraction

Data extraction forms were created for each study and data were extracted independently by the reviewers (AI, AP, LA). Data extracted includes author, publication date, study aims, participant characteristics, medication state, walking surface, walking task and the key findings from the studies, muscles assessed, electrode placement, signal-processing techniques, EMG outcome measures, gait parameters and the gait duration/length analysed. Data were synthesised and formatted into tables. Tables 2 and 3 list aims, participant characteristics, medication state, walking surface, walking task and the key findings from non-intervention and intervention studies, respectively. Tables 4 and 5 contain EMG related descriptors including muscles assessed, electrode placement, signal-processing techniques, EMG outcome measures, gait parameters and the gait duration/length analysed for non-intervention and intervention studies, respectively.

### Quality assessment

A customised quality appraisal form (see Supplementary Information) based on sources addressing the themes in this review was developed. The components of the quality assessment considered both internal and external validity of studies by integrating generic principles of systematic reviews^118^, intervention studies^119,120^, reviews assessing EMG and gait^121,122^ and standardised reporting of EMG data^123,124^.

External validity considers the applicability and generalisability of the study in other settings and contexts. Themes of external validity included participant characteristics and selection methods. Internal validity refers to the extent of no bias in a study validity and other aspects of research design (e.g. randomisation, blinding, study protocol consistency), and the processing of EMG data. The reviewed studies were divided into two groups: intervention and non-intervention. An additional subset of questions was included to assess the quality of intervention studies only. We defined quality of studies as low (<50%), medium (50–69%) and high (≥70%).

## Supplementary information

Supplementary Note 1
