# Supplementary material for: Effect of Parkinson’s disease and two therapeutic interventions on muscle activity during walking: a systematic review
Source: NPJ Parkinsons Dis. 2020 Sep 9;6:22. doi: 10.1038/s41531-020-00119-w (PMC7481232; doi:10.1038/s41531-020-00119-w)
Supplement: Supplementary file 1 — Supplementary Note 1 [file 41531_2020_119_MOESM1_ESM.pdf]

Supplementary Note 1.

## Quality Assessment for EMG Review

The quality of studies included within a review conducting a systematic evaluation of research is commonly assessed by standardised guidelines (1, 2). As these guidelines form a primary focus on healthcare interventions such as randomised controlled trials, the application of these quality assessments to this review was unsuitable for all studies included for review. Thus, a customised quality appraisal form based on sources addressing the themes in this review was developed. The components of the quality assessment considered both internal and external validity of studies by integrating generic principles of systematic reviews (3), intervention studies (1, 2), reviews assessing EMG and gait (4, 5) and standardised reporting of EMG data (6-8).

The external validity considers the applicability and generalisability of the study in other settings and contexts. The internal validity refers to the extent of no bias in a study. The quality assessment was divided into themes of external validity including participant characteristics and selection methods, internal validity including aspects of research design (e.g. randomisation, blinding, study protocol consistency), and the processing of EMG data which can effect subsequent interpretation of results. The reviewed studies were divided into two groups: intervention and non-intervention. An additional subset of questions is included which are applicable to assess the quality of intervention studies only.

**Participant Information**

Selection (External validity)

1. Are the characteristics of the participants in both control and patient groups included in the study clearly described? (Age, gender, UPDRS/H&Y)
2. Are inclusion and exclusion criteria stated?
3. Was a description of identifying the source population provided?
4. Was the sampling method described? (e.g. random, consecutive, opportunity)
5. Were participants representative of the entire population they were recruited from?
6. Were losses of participants or patients at any stage reported and considered?

**Study Information**

Description/specification of study (internal validity)

7. Is the hypothesis/aim/objective of the study clearly described?
8. Are the main outcomes to be measured clearly described in the Introduction or Methods section such that they may be replicated?

**Study Analysis**

9. Is the sample size justified with a power analysis and/or a study referencing a similar sample size?
10. Were the statistical tests used to assess the main outcomes appropriate (i.e. parametric vs. non-parametric)?
11. Have actual probability values been reported (e.g. 0.035 rather than <0.05) for the main outcomes except where the probability value is less than 0.001?
12. Was there adequate adjustment for confounding factors in the analyses from which the main findings were drawn?
13. Are the main findings of the study clearly described?

**EMG Recording and Processing**

14. Was the specific make/ manufacturer of electrode reported or electrode described e.g. type, shape, size, separation?
15. Were electrode locations and orientation over muscle described and justified according to validated methods e.g. SENIAM, ISEK?
16. Was the method of preparing skin and securing the electrodes described?
17. Were the EMG data time-normalised to gait cycle/phase?
18. Were the EMG data amplitude-normalised?
19. Were the hardware and software filters and amplification specified e.g. Filter types, Low and/or high pass cut-off frequencies, slopes of the cut-offs (dB/octave or dB/decade)?
20. Was a validated outcome measure reported and referenced and/or were novel/alternative techniques accompanied by a full scientific description?

**Extra questions to assess intervention studies:**

21. Was the method of subject randomisation into intervention and control group described?
22. Was an attempt made to blind study subjects to the intervention they received?
23. Is the intervention of interest clearly described?
24. Was an attempt made to blind those measuring the main outcomes of the intervention?
25. Have all important adverse events that may be a consequence of the intervention been reported?
26. Was the randomised intervention assignment concealed from both patients and health care staff until recruitment was complete and irrevocable?


1. Downs SH, Black N. The feasibility of creating a checklist for the assessment of the methodological quality both of randomised and non-randomised studies of health care interventions. J Epidemiol Community Health. 1998;52:377–84.
2. Higgins JP, Altman DG, Gotzsche PC, Juni P, Moher D, Oxman AD, et al. The Cochrane Collaboration's tool for assessing risk of bias in randomised trials. BMJ. 2011;343:d5928.
3. Oxman AD. Checklists for review articles. 1994.
4. Rosa MCN, Marques A, Demain S, Metcalf CD. Lower limb co-contraction during walking in subjects with stroke: A systematic review. Journal of Electromyography and Kinesiology. 2014;24(1):1-10.
5. Dobson F, Morris ME, Baker R, Graham HK. Gait classification in children with cerebral palsy: a systematic review. Gait Posture. 2007;25(1):140-52.
6. Merletti R. Standards for Reporting EMG Data. Journal of electromyography and kinesiology. 1999;9(1):3-4.
7. Hermens HJ, Freriks B, Disselhorst-Klug C, Rau G. Development of recommendations for SEMG sensors and sensor placement procedures. Journal of Electromyography and Kinesiology. 2000;10(5):361-74.
8. Soderberg GL, Knutson LM. A Guide for Use and Interpretation of Kinesiologic Electromyographic Data. Physical Therapy. 2000;80(5):485-98.
